# Supplementary material for: Differentiation of mouse fetal lung alveolar progenitors in serum-free organotypic cultures
Source: eLife. 2021 Sep 29;10:e65811. doi: 10.7554/eLife.65811 (PMC8480975; doi:10.7554/eLife.65811)
Supplement: Supplementary file 1. [file elife-65811-supp1.docx]

**Supplementary File 1. Table of qPCR primers**

| **Gene** | **Forward Sequence (5’-3’)** | **Reverse Sequence (5’-3’)** |
| --- | --- | --- |
| *Sox9* | CTGAAGGGCTACGACTGGAC | TACTGGTCTGCCAGCTTCCT |
| *Sox2* | CTCTGCACATGAAGGAGCAC | CTCCGGGAAGCGTGTACTTA |
| *Id2* | CTCCAAGCTCAAGGAACTGG | AGGCTGACGATAGTGGGATG |
| *Etv5* | CGTTGGGGTATCCAGAAGAA | TGCATGATGCCCTTTTCATA |
| *Bmp4* | CGTTACCTCAAGGGAGTGGA | ATGCTTGGGACTACGTTTGG |
| *Epcam* | cctgagagtgaacggagagc | cacaatgacagcgatgatcc |
| *Pdgfra* | cttcggaagagagtgccatc | caccaggtccgaggaatcta |
| *Pecam1* | gcccaatcacgtttcagttt | aaaacgcttgggtgtcattc |
| *Sftpc* | AGCAGACACCATCGCTACCT | GCAGTAGGTTCCTGGAGCTG |
| *Abca3* | TTTCTCATTCCCTCCACCTG | AAGCCGTGCACTCTCATCTT |
| *Lyz2* | GATGGCAAAACCCCAAGAGC | CAGACTCCGCAGTTCCGAAT |
| *Muc1* | GGCCACCACTCCAGTTTACA | TTGACTTGGCACTGAAGGCT |
| *Scnn1a* | CCCTCTGTCACGATGGTCAG | TCCGGAACCTGTGCAGTAAC |
| *Atf3* | GCTGCCAAGTGTCGAAACAA | GTTCCTCTCGTCTTCCGGTG |
| *Ager* | ACGGGACTCTTTACACTGCG | CAACCAACAGCTGAATGCCC |
| *Aqp5* | TCCTGGCTGCAATCCTCTAC | CAGCTCGATGGTCTTCTTCC |
| *Hopx* | ACAAGGTCAACAAGCACCCG | CAGGCGCTGCTTAAACCATT |
| *Pdpn* | TTTGGGGAGCGTTTGGTTCT | CCAGTAGCACCTGTGGTTGT |
| *Akap5* | ACACAGGAAAAGGTCCGAGC | TGACGACTTCTCTTTGCCCC |
| *Cebpa* | GACCCAGAGGATGGTTTCGG | CTCCGCGGAAAGTCTCTCG |
| *Axin2* | TAGGCGGAATGAAGATGGAC | CTGGTCACCCAACAAGGAGT |
| *Actb* | CTCTGGCTCCTAGCACCATGAAGA | GTAAAACGCAGCTCAGTAACAGT |
| *Gapdh* | ACAACTTTGGCATTGTGGAA | GATGCAGGGATGATGTTCTG |
